# Dynamic Prediction of Patient Outcomes in the Intensive Care Unit: A Scoping Review of the State-of-the-Art

**DOI:** 10.1177/08850666231166349

**Published:** 2023-04-05

**Authors:** Linda Lapp, Marc Roper, Kimberley Kavanagh, Matt-Mouley Bouamrane, Stefan Schraag

**Affiliations:** 1 3527 University of Strathclyde, Glasgow, UK; 2 172239 Usher Institute, College of Medicine and Veterinary Medicine, University of Edinburgh, Glasgow, UK; 3 41444 Golden Jubilee National Hospital, UK

**Keywords:** predictive modeling, dynamic prediction, patient outcomes, critical care, intensive care unit

## Abstract

**Introduction:**

Intensive care units (ICUs) are high-pressure, complex, technology-intensive medical environments where patient physiological data are generated continuously. Due to the complexity of interpreting multiple signals at speed, there are substantial opportunities and significant potential benefits in providing ICU staff with additional decision support and predictive modeling tools that can support and aid decision-making in real-time.

This scoping review aims to synthesize the state-of-the-art dynamic prediction models of patient outcomes developed for use in the ICU. We define “dynamic” models as those where predictions are regularly computed and updated over time in response to updated physiological signals.

**Methods:**

Studies describing the development of predictive models for use in the ICU were searched, using PubMed. The studies were screened as per Preferred Reporting Items for Systematic Reviews and Meta-Analysis (PRISMA) guidelines, and the data regarding predicted outcomes, methods used to develop the predictive models, preprocessing the data and dealing with missing values, and performance measures were extracted and analyzed.

**Results:**

A total of n = 36 studies were included for synthesis in our review. The included studies focused on the prediction of various outcomes, including mortality (n = 17), sepsis-related complications (n = 12), cardiovascular complications (n = 5), and other complications (respiratory, renal complications, and bleeding, n = 5). The most common classification methods include logistic regression, random forest, support vector machine, and neural networks.

**Conclusion:**

The included studies demonstrated that there is a strong interest in developing dynamic prediction models for various ICU patient outcomes. Most models reported focus on mortality. As such, the development of further models focusing on a range of other serious and well-defined complications—such as acute kidney injury—would be beneficial. Furthermore, studies should improve the reporting of key aspects of model development challenges.

## Introduction

Many risk prediction models have been developed to assist a range of purposes in healthcare delivery, including hospital bed allocation,^1^ management of medications^2^ and preoperative assessment.^3^ However, risk prediction models for use in intensive care units (ICUs) do not yet harness the full potential of what could be achieved with the optimum use of the rich data sets available in an ICU environment.^4^

Technology-intensive ICUs generate a large volume of patient physiological data that are continuously monitored—and at a higher time-frequency—in comparison to other hospital services. Previous studies have shown that a significant factor in improving clinical outcomes is the timeliness of health interventions,^5,6^ which can be improved with accurate prognosis and early warning. However, commonly used ICU prediction models were often not developed for real-time monitoring but instead as a precalculated risk score not subsequently recomputed according to real-time patient data input.^7^ This means that medical interventions can be initiated as a reactive measure—rather than be planned preventatively—and often only after a complication has already developed.^8^

Hence, this scoping review aims to synthesize the current state-of-the-art in the development and use of dynamic predictive models for the ICU and to provide some future directions for research to improve real-time prediction of patient outcomes in ICU. We considered models to be “dynamic” if predictions are regularly computed and updated over time in response to varying time-dependent physiological signals as opposed to static risk scores computed a priori and not updated with new varying input. This scoping review discusses the outcomes predicted by the models, the algorithms used to develop and compute predictions and their performance. In addition, how models mitigate and manage known data processing challenges, such as missing data or imbalanced classification issues are discussed in Supplemental Material.

There have been no reviews conducted to date on the prediction of patient outcomes in the ICU in real-time and thus the present work makes an important contribution to the advancement of the state-of-the-art as well as informing future directions of research in this field. This paper shows that there are numerous models developed to predict patient outcomes in a dynamic manner for the use of the ICU. However, further detail in the model development process is needed to provide transparency and allow for validation.

## Methods

PubMed was searched for relevant articles published between 1 January 2000 and 25 April 2022. An updated search was conducted of relevant articles published between 26 April 2022 and 23 January 2023. In addition, references from included studies were further screened for potential additional relevant studies. We followed a scoping review methodology first advocated by Arksey and O'Malley^9^ and further refined by Levac et al^10^

The review was conducted, following the Preferred Reporting Items for Systematic Reviews and Meta-Analysis (PRISMA) guidelines, modified for scoping reviews.^11^ The reviewing team included experts in anesthesia, cardio-thoracic surgery, and ICUs (SS), digital health, decision support and scoping reviews (MMB) and biostatistics and machine learning (LL, KK, MR).

Scoping reviews can be used to identify emerging patterns in the literature for potentially extremely large research domains such as “digital technologies for postoperative care”, for example.^12^ This mode of investigation is particularly useful for researching emerging technology innovations, where identifying broad trends is key as is the case for this study.

### Data Sources and Search Strategy

The following search query was conducted on PubMed:

((dynamic predict*) OR (real time predict*)) AND ((patient outcome*[Title/Abstract]) OR (mortality[Title/Abstract]) OR (morbidity[Title/Abstract]) OR (complication*[Title/Abstract])) AND ((critical care) OR (intensive care)) NOT (cancer) NOT (COVID-19) NOT (Paediatric) NOT (Paediatric) NOT (trauma).

The titles and abstracts of the articles retrieved in the search were screened as to the eligibility criteria further described in “Eligibility Criteria and Analysis” section.

### Data Extraction

A data extraction instrument was developed by 1 researcher (LL) and discussed with the investigative team before being piloted with a sample of selected studies until a final version of the instrument was agreed upon by consensus. The data extracted for included studies included: Study authors, year of publication, patients included in the study, predicted outcomes, methods used to develop the predictive models, methods to preprocess the data and deal with missing values, types of features used in the model and performance measures used in the study. Mendeley^13^ was used for citation management.

### Eligibility Criteria and Analysis

We define “dynamic” models as those where predictions are regularly computed and updated over-time in response to varying time-dependant physiological signals or updated input (such as repeated laboratory tests, for example).

The frequency of the input can be variable depending on the nature of the data source: eg, every second, minute or hour, or even less often (eg, daily for a laboratory test). The common aspect across the range of included models however is that these models are designed to update their prediction results as new data is input over time.

The inclusion and exclusion criteria are described below and in Table 1*.* Based on the inclusion criteria, in terms of study design, we included studies on the development of dynamic risk prediction models used in ICU. Only adult critical care or ICU patients were included in the study. In terms of the predicted outcome, only studies including classification tasks were included. This decision was made because usually adverse clinical outcomes, such as mortality or complications, are defined as binary categorical outcomes, or are diagnosed based on a number of laboratory variables, as opposed to 1 numerical variable.^14^ Studies describing the development of prediction models for outcomes directly related to patient health were included, including *mortality, complications*, and *ICU stay.* Studies describing prediction models that were developed using repeated measures of laboratory test results were included in the review. These could be laboratory results that are measured every hour or every day. Also, studies describing the use of vital signs that were frequently measured (eg, every second or every minute) were also included. Finally, only studies that reported the models’ performance measures were included in the review.

**Table 1.** Inclusion and Exclusion Criteria for Studies Based on Patients Included in the Study, Variables Used in Analysis, Outcome of the Analysis, Intervention, and Study Design.

Criterium	Included	Excluded
Study design	Primary study, ie, study that develops a prediction model	Review article, validation study, commentary.
Patients	Adult critical care or intensive care patients, noncancer patients, nonCOVID-19 patients, nontrauma patients	Any other patient who is not admitted to critical or intensive care, cancer patients, COVID-19 patients, trauma patients.
Setting	Adult critical care or intensive care unit	Pediatric critical care or intensive care unit, emergency department, hospital wards, or any other hospital setting that is not adult critical care or intensive care unit.
Type of a problem	Classification	Regression, or any other method that is not classification
Outcome	Patient outcomes: mortality, morbidity, postoperative complications, hospital length of stay, ICU length of stay or any other outcome that is directly related to patient's health	Outcomes that are not directly related to the patient (eg, costs)
Variables	Includes laboratory data that were treated as dynamic variables	Includes only static variables (ie, that are measured once) or variables that are not vital signs or laboratory data
Type of model	Must be a model predicting patient outcomes based on dynamic variables on a “real-time” basis.	Static prediction model
Comparator	Any model performance measure (eg, AUC, sensitivity, specificity, accuracy, etc)	No model performance reported

Abbreviations: AUC, area under the curve; ICU, intensive care unit; COVID-19, coronavirus disease 2019.

Based on the exclusion criteria, studies focusing on the evaluation of models—but not including a description of the model development—or reviews of models were excluded. Prediction models developed specifically for cancer or trauma patients were excluded. While predicting ICU outcomes for cancer or trauma patients could help with managing unplanned ICU admissions,^15^ the additional confounding variables that cancer or trauma could add are most likely not relevant for general ICU patients. Since the coronavirus disease 2019 (COVID-19) pandemic from early 2020, many prediction models have been developed to predict COVID-19-related outcomes.^16^ Because, this is a nonroutine situation, studies with COVID-19 patients were excluded. The models could include static variables (measured only once) to aid prediction, however, studies that used only static variables that were calculated for an *a priori* risk score were excluded.

### Study Selection

**Figure 1.**
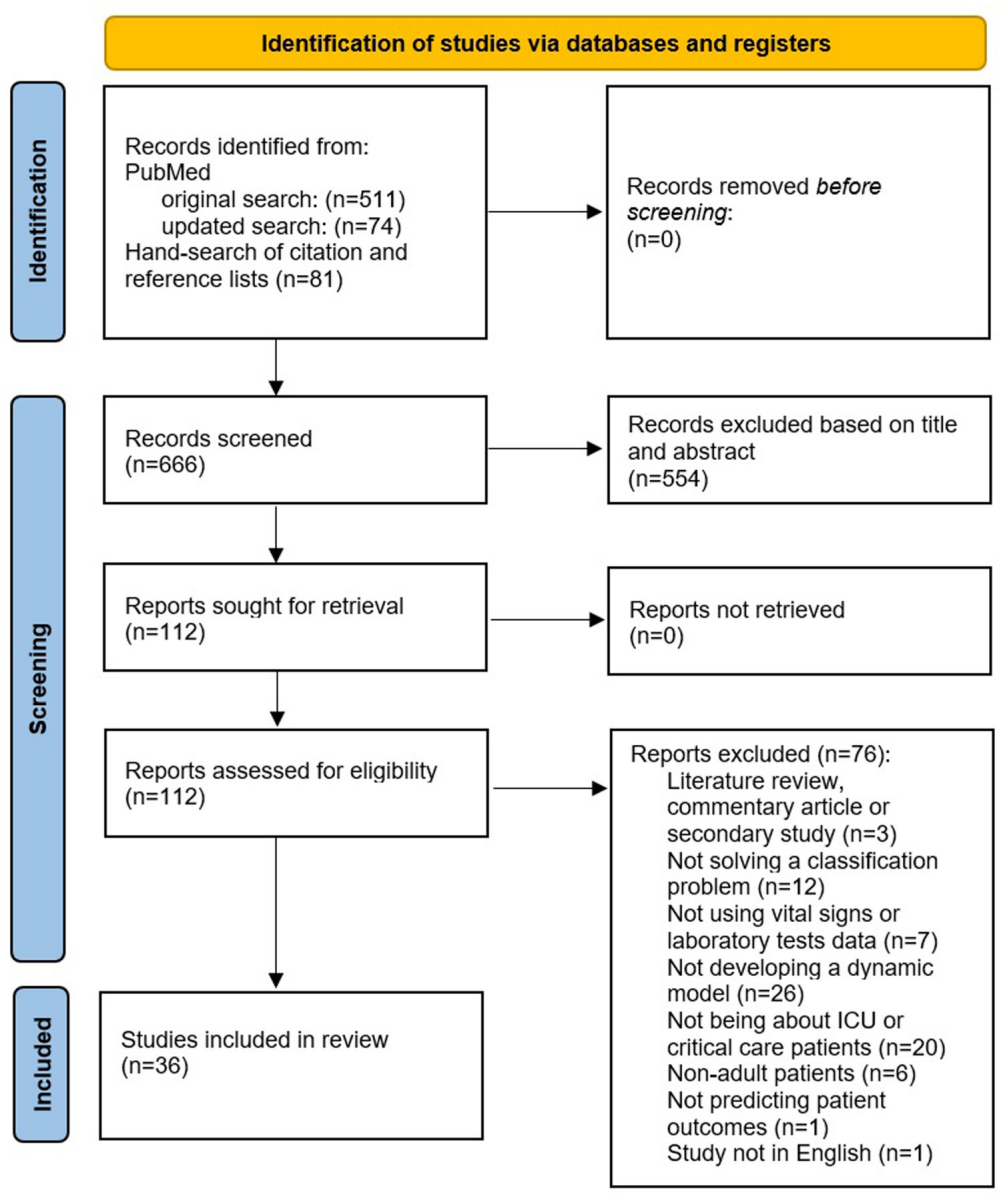
Preferred Reporting Items for Systematic Reviews and Meta-Analysis (PRISMA) flow diagram outlining the selection process for identifying studies included in the final review.

The initial search query on PubMed retrieved n = 511 articles (see PRISMA flow-chart in Figure 1). In addition, n = 81 studies were identified from manual searching of citations and the reference lists of included studies. The updated search from PubMed retrieved n = 74 additional papers. This resulted in a total of n = 666 records screened based on the title and abstract. n = 554 studies were excluded at the initial abstract screening stage due to not meeting the eligibility criteria (ie, not being a primary study, concerning nonadult patients outside of critical care or ICU, not predicting patient outcomes), which left n = 112 articles for the full screening stage. After full-text screening, n = 36 articles were included in the final review for data extraction and synthesis. The reasons for why the records were excluded from the full screening stage are shown in Figure 1.

## Results

### Included Studies

Thirty-six studies were included in the review.^17–52^ The data extraction table for these studies is described in Table 2. The majority of the studies were conducted in the USA (n = 20 studies), n = 6 studies were conducted in China, and n = 2 in India. Other countries represented in the review include Australia, Germany, Finland, the Netherlands, Portugal, South Korea, Thailand, and the UK.

**Table 2.** Data Extraction Table for the Included Studies.

First Author	Year	Country	Number of Patients	Patient Population	Outcome Predicted	MIMIC	eICU	Data Types	Types of Variables
Bhattacharya	2018	India	4547 patients	All ICU adult patients	Acute hypotensive episode	MIMIC-II	No	Numerical	5 vital signs
Caballero	2015	USA	11 648 patients	All ICU patients	Mortality	MIMIC-II	No	Mixed	Vital signs, laboratory results, medical notes
Deasy	2020	UK	46 476 patients	All adult patients admitted to critical care who stayed in hospital for > 48 h	Mortality	MIMIC-III	No	Mixed	Patient demographics, vital signs, laboratory tests
Dummitt	2018	USA	7819 patients	All ICU patients	Septic shock	No	No	Mixed	Patient demographics and vital signs
Feng	2021	China	5653 patients	Adult patients with noninvasive ventilation for over 48 h in ICU	Noninvasive ventilation failure	MIMIC-III	No	Mixed	Patient demographics, vital signs, laboratory tests
Ghosh	2017	Australia	1310 patients	ICU patients	Septic Shock	MIMIC-II	No	Numerical	Mean arterial pressure, heart rate and respiratory rate
Gultepe	2014	USA	741 patients	ICU patients with systemic inflammatory response syndrome	Mortality, lactate level (Sepsis)	No	No	Numerical	Vital signs and laboratory results
Henry	2015	USA	16 025 patients	All ICU patients, any surgery	Septic shock	MIMIC-II	No	Mixed	Patient demographics, vital signs, and laboratory results
Hernandez	2021	USA	406 patients	Adult patients in ICU recovering from surgery	Hemodynamic instability	No	No	Mixed	Patient demographics, vital signs, and ECG data
Hu	2022	China	2170 patients	Adult medical ICU patients	“Life-threatening events”, ie, any sign of cardiac arrest	No	No	Mixed	Patient demographics, vital signs, laboratory results
Huddar	2016	India	775 patients	ICU patients	Acute respiratory Failure	MIMIC-II	No	Mixed	Clinical Notes + vital signs
Hug	2009	USA	10 066 patients	Adult ICU patients	Mortality	MIMIC-II	No	Mixed	Patient demographics, vital signs, and laboratory results
Johnson	2017	USA	50 488 patients	ICU patients corresponding to adults for surgical, medical, neurological and coronary critical illness	Mortality	MIMIC-III	No	Mixed	Patient demographics, vital signs, and laboratory results
Joshi	2012	USA	10 000 patients	Adult patients in ICU	Mortality	MIMIC-II	No	Mixed	Vital signs and laboratory results
Lee	2010	USA	1311 patients	ICU patients	Hypotensive episodes	MIMIC-II	No	Mixed	Vital signs
Lehman	2015	USA	453 patients	ICU patients	Mortality	MIMIC-II	No	Numerical	Vital signs
Lehman	2013	USA	337 patients	ICU patients with day 1 SAPS-I scores and at least 18 jours of blood pressure data since 24 h from ICU admission	Mortality	MIMIC-II	No	Numerical	Vital signs
Luo	2022	China	15 603 patients	Adult ICU patients with sepsis-associated acute kidney injury	Mortality	MIMIC-IV (development and internal validation)	Yes (External validation)	Mixed	Patient demographics, vital signs, laboratory tests
Ma	2019	USA	3763 patients	Medical ICU patients	Mortality	No	No	Mixed	Patient demographics, vital signs, laboratory tests
Mao	2012	USA	772 patients	ICU patients	Mortality	MIMIC-II	No	Numerical	Vital signs
Meyer	2018	Germany	11 492 patients (development), 5898 (external validation)	All ICU patients, Any surgery	Bleeding, mortality and renal failure	MIMIC-III (external validation)	No	Mixed	Patient demographics, vital signs, and laboratory results
Misra	2021	USA	45 425 patients	Adult ICU patients	Septic shock	No	No	Mixed	Patient demographics, vital signs and laboratory results
Mohammed	2020	USA	5958 patients	Adult medical ICU patients	Sepsis	No	No	Numerical	Vital signs
Nemati	2018	USA	27 527 (development), 42 411 (external validation)	All ICU patients, regardless of reason being there	Sepsis	MIMIC-III (external validation)	No	Mixed	Patient demographics, vital signs and laboratory results
Park	2020	South Korea	36 023 patients	Adult ICU and Ward patients	Bacteremia	No	No	Mixed	Patient demographics, vital signs and laboratory data
Pattalung	2021	Thailand	18 353 MIMIC-III patients, 18 134 MIMIC-IV patients, 36 283 eICU patients	Adult ICU patients staying in ICU for > 48h	Mortality	MIMIC III and MIMIC-IV	Yes	Numerical	Vital signs and laboratory variables
Raj	2019	Finland	472 patients	Adult traumatic brain injury patients	Mortality	No	No	Numerical	Patient demographics, vital signs, and laboratory results
Ryan	2022	USA	4267 patients	Adult ICU patients undergoing cardiac surgery	Acute kidney injury	MIMIC-IV	No	Mixed	Demographics, vital signs, laboratory variables, preoperative, intraoperative variables
Shashikumar	2017	USA	242 patients	Adult ICU patients	Sepsis	No	No	Mixed	Patient demographics and vital signs
Silva	2006	Portugal, 42 ICUs of 9 EU countries (list of countries unavailable)	13 164 patients	Adult patients in ICU that did not have burns or had a bypass surgery	Mortality	No	No	Numerical	17 variables collected within the first 24 h of admission
Thoral	2021	The Netherlands	14 105 admissions	Adult ICU patients	ICU readmission and mortality as a composite outcome	No	No	Mixed	Patient demographics, vital signs and laboratory results
van Wyk	2019	USA	754 patients	All ICU adult patients	Sepsis	No	No	Numerical	Vital signs and laboratory results
Xia	2019	China	18 415 patients	Adult ICU patients with length of stay >10 days	Mortality	MIMIC-III	No	Numerical	Vital signs and laboratory results
Yee	2019	USA	9165 patients	All ICU patients	Septic shock	MIMIC-III	No	Mixed	Patient demographics and laboratory results
Yijing	2022	China	1860 patients	Adult ICU patients	Cardiac arrest	MIMIC-III	No	Numerical	ECG, and vital signs
Zhao	2021	China	11 362 patients (development), 35 252 (external validation)	Adult ICU patients who stayed in ICU >24h	Sepsis-induced coagulopathy	MIMIC-IV (development)	Yes (external validation)	Mixed	Patient demographics, vital signs, and laboratory results

Abbreviations: MIMIC, multiparameter intelligent monitoring in intensive care; ICU, intensive care unit.

In terms of study size, 5 studies used more than 30 000 patient records in the development of their models^20,28,29,33,53^; with Johnson et al^20^ using the largest number of patients of 50 488 in their model development. Nine studies used between 10 000 and 30 000 patient records.^18,19,27,31,36,37,39,43,46,50^ Nine studies use considerably smaller datasets of less than 1000 patient records,^8,23,24,26,34,35,38,49,51^ with the smallest study population being the study by Shashikumar et al (242 patient records).^35^

External validation was carried out by 4 studies.^27,31,43,46^ When most studies were single-center studies, it is worth noting that Silva et al^36^ used data from 42 ICUs from 9 European Union countries.

In terms of the data used, out of 36 studies included in this review, 21 of them developed their models using data from a version of the Multiparameter Intelligent Monitoring in Intensive Care (MIMIC) database.^54^ Eleven studies used the MIMIC-II,^17–19,21–24,26,48,50,52^ 6 studies used the MIMIC-III,^20,29,39,41,42,47^ and 4 studies used the MIMIC-IV.^33,43,45,46^ Two studies used the MIMIC-III for validating their models externally.^27,31^ Of publicly available datasets, 3 studies also used the eICU database^55^—1 for development of the model^33^ and 2 for external validation.^43,46^

### Specific Outcomes Predicted by the Models

As shown in Table 3, the most commonly predicted outcome was mortality (n = 17 studies), while n = 12 studies predicted sepsis, n = 5 studies predicted cardiovascular complications, and n = 5 studies predicted other types of complications, such as respiratory, renal complications and bleeding. When comparing the models among studies included in this review, it is also important to bear in mind that the predicted outcomes can have a range of definitions across studies.

**Table 3.** Outcomes Predicted by Included Studies.

Outcome	Number of Studies	Author and Year	Prevalence of the Outcome
Mortality	17	Caballero 2015	Not reported
		Deasy 2020	13.0%
		Gultepe 2014	35.0%
		Hug 2009	Not reported
		Johnson 2017	Not reported
		Joshi 2012	12.0%
		Lehman 2013	14.0%
		Lehman 2015	15.0% - 19.0%*
		Luo 2022	17.0% - 18.6%*
		Ma 2019	1.2%—17.0%*
		Mao 2012	2.3%
		Meyer 2018	6.2%
		Pattalung 2021	8.0%—14.1%*
		Raj 2019	19.5%
		Silva 2006	Not reported
		Thoral 2021	5.3%
		Xia 2019	11.7%
Sepsis	12	Dummitt 2018	2.3%
		Ghosh 2017	15.9%
		Gultepe 2014	20.3%
		Henry 2015	14.1%
		Misra 2021	12.7%
		Mohammed 2021	10.35%
		Nemati 2018	8.6%
		Park 2020	1.9%-2.3%*
		Shashikumar 2017	22.0%
		van Wyk 2019	32.5%
		Yee 2019	1.9%
		Zhao 2021	59.0%
Cardiovascular complications	5	Bhattacharya 2018	28.5%
		Hernandez 2021	35.0%
		Hu 2022	15.3%
		Lee 2010	24.2%-25.4%*
		Yijing 2022	9.1%
Respiratory complications	2	Feng 2021	46.7%
		Huddar 2016	11.7%
Bleeding	1	Meyer 2018	4.9%
Renal complications	2	Meyer 2018	1.0%
		Ryan 2022	50.0%

*If a range is reported, the authors carried out different experiments with different datasets, where the prevalence of outcome varied.

#### Mortality

Most models predicting mortality focused on short-term mortality, specifically happening while in the ICU.^18,20,21,23,29,33,36,39,49,56^ In Hug et al^19^ the focus was broader however as it predicted the risk of both death while in the ICU but also within 30 days of discharge from the ICU. The 4 models of Lehman et al, Luo et al, Mao et al and Meyer et al focused on prediction of in-hospital mortality.^24,26,27,46^ Finally, Raj et al focused on the prediction of 30-day mortality^34^ and Thoral et al^37^ on a composite outcome of in-hospital mortality and ICU readmission within 7 days of ICU discharge.

The prevalence of mortality in the studies ranged vastly, ie, between 1.2% and 35.0%. This is due to the studies using data from different types of patient populations, where some had an increased risk of mortality than others. For example, Ma et al^25^ found that among medical ICU patients, 1.2% of patients died within 6 h of ICU admission. Gultepe et al,^49^ however predicted mortality among sepsis patients where a very high mortality rate (35.0%) was expected.

#### Sepsis

A large study investigating sepsis-related mortality in English ICUs found that sepsis can affect a quarter of adult ICU patients in England, and can kill 1 in 4 ICU patients affected.^57^ Sepsis occurs when an infection in the body results in a systemic inflammatory response syndrome and is defined to be severe if sepsis causes organ dysfunction.^58^ Sepsis can have a significant impact on patients due to being associated with increased mortality and life-long complications, such as permanent organ damage, cognitive impairment, and physical disability.^58^

The definition of sepsis varied substantially among studies. Four studies^30,31,35,43^ used the Third International Consensus Definitions for Sepsis and Septic Shock (Sepsis-3) criteria^14^ to predict the onset of sepsis in general. Two studies^28,40^ used the Systemic inflammatory Response Syndrome (SIRS) criteria^59^ to predict specifically septic shock. It is worth mentioning that Misra et al^28^ treated septic shock patients as the cases and patients with other sepsis-related complications as controls. The studies by Ghosh et al^48^ and Henry et al^50^ defined septic shock as the outcome in a similar way as SIRS criteria, however, they did not specifically state that they were using this widely used, agreed upon criteria. Van Wyk et al^38^ predicted the onset of sepsis by following the Sepsis-2 criteria,^58^ even though a new criteria (Sepsis-3) had already been published 3 years prior the van Wyk et al's study. This is a limitation to van Wyk et al's study as later published studies showed that the definition of sepsis by these 2 criteria was very different,^60^ and hence the developed models can misclassify patients to have different levels of sepsis.

Avoiding the conflicting sepsis definition criteria, Gultepe et al^49^ predicted high lactate levels (≥ 4 mmol/l vs <4 mmol/l), which is considered to be a sign of possible sepsis. Yee et al,^41^ however, made their own criteria for septic shock. This is considered to be a limitation to the study as, even though the currently available criteria for diagnosis of septic shock are not perfect,^61^ they are still based on consensus and are heavily validated.^62,63^ Finally, Park et al^32^ predicted bacteremia, which is a form of sepsis, and hence was included under this category in this review.

Similarly to the models predicting mortality, the prevalence of sepsis also varied substantially: 1.9%–59.0%. Yee et al^41^ predicted septic shock in the whole ICU population, resulting in a very low prevalence of septic shock of 1.9%. Park et al^32^ predicted bacteremia also in the general ICU, resulting with low prevalence (between 1.9% and 2.3%, depending on experiment). Zhao et al,^43^ however, analyzed sepsis patients only, and predicted sepsis-induced coagulopathy, which turned out to be very prevalent (59.0%) among sepsis patients.

#### Cardiovascular complications

Two models predicted hypotensive episodes.^17,22^ Acute hypotensive episode is a sudden onset of a period of sustained low blood pressure.^17^ Bhattacharya et al defined hypotensive episode as a period of 30 min where at least 90% of mean arterial pressure measurements were no greater than 60 mmHg. A long-lasting hypotension can result in dangerously decreased tissue blood flow with consequent end-organ damage. Treating hypotension appropriately can be effective to avoid severe sepsis,^64^ shock^65^ and acute coronary syndrome.^66^

Hernandez et al^51^ predicted hemodynamic instability, which is related to arrhythmia, respiratory failure and hypotension. They did not clearly define, however, what they considered hemodynamic instability to be.

Hu et al^44^ predicted “lie-threatening” events, defined as “all cardiac arrest-related cardiopulmonary resuscitation”, among medical ICU patients.

Yijing et al^42^ predicted cardiac arrest in critically ill patients. The cardiac arrest was defined as the start time of the first occurrence of the specified abnormal events, however the abnormal events were not described in the study. This is a limitation to the study as it makes the prediction model difficult to reproduce.

When looking at studies predicting various cardiovascular complications, the prevalence was also very variable: between 9.1% and 35.0%. This is because hypotensive episodes and hemodynamic instability are more common complications, especially in cardiac patients, who were included in Hernandez et al's, Bhattacharya's, and Lee's datasets, resulting in a high number of patients with the predicted outcomes.^17,22,51^ Cardiac arrest, however, is a less common complication, especially if all ICU patients are included in the dataset, not only cardiac surgery patients.^67^ Hence, Yijing et al^42^ predicted an outcome that had a prevalence of 9.1% in their study population.

#### Other complications

Feng et al^47^ predicted late noninvasive ventilation failure in ICU. They defined the outcome as death during or intubation after noninvasive ventilation. Interestingly, in Feng et al's^47^ patient cohort, the prevalence of late noninvasive ventilation failure was very high (46.7%). This could be because they included patients who received noninvasive ventilation as a primary treatment following ICU admission.

Huddar et al^8^ predicted acute respiratory failure, which occurs when the respiratory system fails in oxygenation and/or CO_2_ elimination from the lungs. It is considered to be the end point of respiratory complications, such as pneumonia or atelectasis. There are various factors than can be associated with acute respiratory failure: patient-related factors, including age, preexisting chronic obstructive pulmonary disease, congestive heart failure, and arrhythmia; and procedure-related variables, including emergency surgery, prolonged surgery, and surgical site.^68^ Compared to the other studies, Huddar et al reported the common incidence of acute respiratory failure ranging between 0.2% and 3.4%, however, in Huddar et al's patient population, the incidence of acute respiratory failure was 11.7%. This might be because Huddar et al retrospectively diagnosed the complication based on a specific criterion that followed the vital signs recorded automatically in the ICU,^8^ whereas studies in the literature are using different definition of what constitutes respiratory failure in a patient.^69^ This shows that some complications that are reported without specific criteria based on laboratory results or vital signs can be under-reported in the electronic health records.

In addition to mortality, Meyer et al^27^ also predicted postoperative bleeding and renal failure requiring renal replacement therapy. The renal failure was defined using Kidney Disease: Improving Global Outcomes (KDIGO) criteria.^70^ Acute kidney injury, formerly called acute renal failure, is a sudden decline in glomerular filtration rate.^71^ Glomeruli are tiny filters in the kidneys that filter waste from the blood. This rate estimates how much blood passes through the glomeruli each minute. Acute kidney injury is usually caused by an event that leads to kidney malfunction, such as dehydration, blood loss from major surgery or injury, or the use of medicines.^72^

Even though acute renal failure in cardiac patients is often relatively low,^73^ Meyer et al's^70^ prevalence for renal failure was very low (1.0%). This might be due to different studies defining acute renal failure differently. Meyer et al, however, used the KDIGO criteria, which is an internationally recognized criteria for diagnosing renal complications, including renal failure.

Finally, Ryan et al^45^ also predicted acute kidney injury among cardiac patients, where the complication was also defined by KDIGO criteria. Interestingly, postoperative stage 1 AKI was diagnosed in 50% of the patients, which is a very high prevalence. While this was not mentioned in their paper, this might indicate that the patient population was chosen to be balanced in terms of the prevalence of the outcome of interest.

### Classification Methods Used by Studies to Predict Patient Outcomes in a Dynamic Manner

As shown in Table 4, the most used methods were logistic regression (20 studies), random forest (13 studies), support vector machines (11 studies), and neural networks (11 studies). Other more commonly used methods included gradient boosting machines (8 studies), and naïve Bayes (4 studies).

**Table 4.** Classification Methods Used by Studies to Predict Patient Outcomes Dynamically.

Method	Number of Studies	First Author and Year
Logistic regression (all versions)	20	Caballero 2015
		Dummitt 2018
		Feng 2021
		Hu 2022
		Huddar 2016
		Hug 2009
		Johnson 2017
		Joshi 2012
		Lehman 2013
		Lehman 2015
		Mao 2012
		Misra 2021
		Raj 2019
		Ryan 2022
		Shashikumar 2017
		Silva 2006
		Thoral 2021
		van Wyk 2019
		Zhao 2021
Random forest	13	Caballero 2015
		Dummitt 2018
		Feng 2021
		Hernandez 2021
		Hu 2022
		Huddar 2016
		Ma 2019
		Misra 2021
		Mohammed 2020
		Ryan 2022
		Thoral 2021
		van Wyk 2019
		Zhao 2021
Support vector machines	11	Ghosh 2017
		Gultepe 2014
		Hernandez 2021
		Hu 2022
		Huddar 2016
		Mao 2012
		Misra 2021
		Mohammed 2020
		Thoral 2021
		van Wyk 2019
		Zhao 2021
Neural Networks (any kind)	11	Deasy 2020
		Meyer 2018
		Park 2020
		Pattalung 2021
		van Wyk 2019
		Feng 2021
		Lee 2010
		Ryan 2022
		Silva 2006
		Feng 2021
		Xia 2019
Gradient boosting machine (all versions)	8	Feng 2021
		Hu 2022
		Johnson 2017
		Luo 2022
		Ryan 2022
		Thoral 2021
		Yijing 2022
		Zhao 2021
Naïve Bayes	4	Caballero 2015
		Gultepe 2014
		Hernandez 2021
		Zhao 2021
Cox proportional hazards	3	Dummitt 2018
		Henry 2015
		Nemati 2018
Decision trees	3	Huddar 2016
		Misra 2021
		Zhao 2021
AdaBoost	2	Hernandez 2021
		Huddar 2016
Bayesian networks	2	Gultepe 2014
		Yee 2019
Hidden Markov models	2	Ghosh 2017
		Gultepe 2014
C5.0	1	Misra 2021
CatBoost	2	Ryan 2022
		Zhao 2021
Dual boundary classifier	1	Bhattacharya 2018
Gaussian mixture model	1	Gultepe 2014
LASSO	1	Johnson 2017
LUCCK (Learning using concave and convex kernels)	1	Hernandez 2021

To take the serial nature of the data into account, the studies had different approaches. It was common to use summary statistics, such a mean or median, minimum and maximum, first and last values within predetermined time windows^19,20,28,31,33,34,36–38,42–46^. For some models, the classification method handled the time-series data.^17,22,27,29,32,39,41,50^ However, some studies used special methods to include the temporal aspect into their model.^18,21,23–26,30,40,47–49,51,52,74^

For example, Caballero et al^18^ used Kalman filtering equations to update the outcome when time-series observations became available. Gultepe et al^49^ used Bayesian network structure learning to capture the time-series aspect of their data. Joshi et al^21^ used radial domain folding to summarize patient state for each time window, which was then included in their prediction model. Ma et al^25^ fitted continuous trajectory to each time series, which was then summarized, using splines, resulting in coefficients that were used to capture information about the shape of the time series.

The outcomes were predicted in varying frequencies. The closest to “real-time” models were those that updated their prediction every time new measurements were entered into the system. Eleven studies followed this prediction frequency.^8,17,26,27,36,39,41,42,48,50,51^ Eight studies developed models to predict outcomes on an hourly basis.^18,20,22–24,29,33,37,45^

Twelve studies predicted the outcomes less often.^25,28,30–32,34,35,38,40,43,44,46,47,49^ More specifically, Ma et al^25^ predicted mortality every 6 h. Nemati et al^31^ predicted sepsis 12, 8, 6 and 4 h before the onset. Park et al^32^ predicted bacteremia 8, 16 and 24 h in advance. Raj et al^34^'s model made new predictions of mortality every 8 h. For Shashikumar et al's^35^ model, sepsis was predicted 4 h in advance. Dummitt et al^40^ made the prediction of septic shock 4, 8, and 24 h beforehand. Feng et al's^47^ model predicted late noninvasive ventilation failure in 8, 16, 24, 36 and 48 h after the start of noninvasive ventilation. Gultepe et al^49^ predicted mortality and high lactate levels in 6, 12, and 24 h. Misra et al^28^ predicted septic shock within 1, 3, and 6 h before the onset. Mohammed et al^30^ predicted sepsis at around 18 h beforehand. Van Wyk et al^38^ predicted sepsis 3 and 6 h in advance. Zhao et al, Luo et al and Hu et al predicted patient outcomes on a daily basis.^43,44,46^

For 2 studies it was unclear how often their dynamic models predicted the outcomes.^19,21^

Information about handling missing data and the imbalanced classification problem were also synthesized from the paper. The most common methods for handling missing data were imputation methods. The rate of missing data in studies was not very well documented and a third of the included studies did not report how missing data were handled, which is a clear indication for lack of transparency.

A third of the studies were dealing with highly imbalanced classification problems, where the prevalence of the predicted outcome was <10%. While it is known that balancing methods or developing models on training sets that have a balanced outcome can lead to poor calibration, where the probability of the predicted outcome is overestimated,^75^ n = 7 studies used balancing methods like Synthetic Minority Oversampling Technique (SMOTE) and upsampling.

The detailed findings and discussion regarding handling missing data and the imbalanced classification problem can be found from the Supplemental Material.

### Performance of the Models

Several studies tested various methods to predict patient outcomes, however, Tables 5–7 show the highest performing models and their respective performance measures for the studies.

**Table 5.** Best-Performing Classification Method and Their Respective Highest Reported Performance of Studies Predicting Mortality.

Author and Year	Classification Method	Accuracy	AUROC	Sensitivity	Specificity	PPV	NPV	AUPRC	F1 Score
Caballero 2015	Logistic regression		0.866	0.789	0.791				
Deasy 2020	Recurrent neural network		0.770						
Gultepe 2014	Support vector machine	0.728	0.726	0.949	0.308				0.821
Hug 2009	Logistic regression		0.885						
Johnson 2017	Gradient boosting machine		0.920					0.665	
Joshi 2012	Logistic regression		0.890						
Lehman 2013	Logistic regression		0.800						
Lehman 2015	Logistic regression		0.700						
Luo 2022	XGBoost	0.866	0.848	0.600	0.879				
Ma 2019	Random forest		0.905					0.381	
Mao 2012	Support vector machine		0.633	0.143	0.950	0.415	0.791		
Meyer 2018	Recurrent deep neural network	0.880	0.950	0.850	0.910	0.900	0.860		
Pattalung 2021	Recurrent neural network		0.910	0.810	0.860	0.850	0.820		
Raj 2019	Logistic regression		0.840						
Silva 2006	Artificial neural network	0.792	0.871	0.781	0.795				
Thoral 2021	Gradient boosting machine		0.789					0.202	
Xia 2019	Long-short term memory	0.753	0.845	0.776	0.750	0.294		0.486	0.426

**Table 6.** Best-Performing Classification Method and Their Respective Highest Reported Performance of Studies Predicting Sepsis.

Author, Year	Classification Method	Accuracy	AUROC	Sensitivity	Specificity	PPV	NPV	AUPRC	F1 Score
Dummitt 2018	Generalized linear model via penalized maximum likelihood		0.860						
Ghosh 2017	Coupled hidden Markov models	0.871							
Gultepe 2014	Gaussian mixture model	0.843	0.849	0.928	0.500				0.905
Henry 2015	Cox proportional hazards		0.830	0.850	0.670				
Misra 2021	Random forest		0.948	0.839	0.881				
Mohammed 2021	Random forest	0.768		0.739	0.796	0.788			0.760
Nemati 2018	Weilbull-Cox proportional hazards	0.670	0.850		0.670				
Park 2020	Recurrent neural network		0.960	0.940					
Shashikumar 2017	Elastic net logistic classifier		0.780	0.850	0.550				
van Wyk 2019	Random forest			0.800					0.680
Yee 2019	Bayesian network		0.810	0.790	0.660	0.460	0.900		
Zhao 2021	Categorical boosting		0.869	0.820	0.757				

**Table 7.** Best-Performing Classification Method and Their Respective Highest Reported Performance of Studies Predicting Respiratory, Cardiovascular, Bleeding, and Renal Complications.

Author and Year	Classification Method	Accuracy	AUROC	Sensitivity	Specificity	PPV	NPV	AUPRC	F1 Score
Respiratory complications									
Feng 2021	Time updated light gradient boosting machine		0.912						
Huddar 2016	Support vector machine		0.873						
Cardiovascular complications									
Bhattacharya 2018	Dual boundary classifier	0.870		0.830	0.900				
Hernandez 2021	Random forest		0.890						
Hu 2022	Light gradient boosting model		0.905	0.763	0.872	0.081			
Lee 2010	Artificial neural network	0.758	0.819	0.748	0.746	0.665	0.833		
Yijing 2022	Extreme gradient boosting	0.960	0.940	0.860	0.850				
Bleeding									
Meyer 2018	Recurrent deep neural network	0.800	0.870	0.740	0.860	0.840	0.770		
Renal complications									
Meyer 2018	Recurrent deep neural network	0.900	0.960	0.940	0.860	0.870	0.940		
Ryan 2022	Ensemble model	0.860	0.950						

Abbreviations: PPV, positive predictive value; NPV, negative predictive value.

#### Mortality prediction

When looking at how the models performed based on predicting mortality (Table 5), Meyer et al^27^ had the highest AUROC of 0.950 when predicting mortality, achieved with recurrent deep neural network. The second-best performance was achieved by Johnson et al^20^ with the AUROC of 0.920 (gradient boosting machine), followed by Pattalung et al^33^ (AUROC = 0.910, recurrent neural network) and Ma et al^25^ (AUROC = 0.905, random forest).

In terms of sensitivity, the model by Gultepe et al^49^ has by far the highest sensitivity of 0.949, achieved with support vector machine. The model developed by Mao et al^26^ has the highest specificity of 0.950 (support vector machine). Based on the accuracy, Meyer et al^27^ had the highest performance of 0.880 (recurrent deep neural network), and they also achieved very high positive predictive value (PPV) and negative predictive value (NPV) of 0.900 and 0.860, respectively. Only 4 studies reported AUPRC when predicting mortality, Johnson et al^20^ with the highest of 0.665 (gradient boosting machine), and out of the 2 studies that reported the F1 score, Deasy et al^29^ achieved the highest of 0.821 with recurrent neural network.

In terms of calibration, Hug et al, Ma et al use Homer-Lemeshow test to assess calibration. Hug et al^19^ found that their model's calibration is weak, whereas Ma et al^25^ found their model to be well-calibrated. Raj et al^34^ found that their algorithm overestimates the risk of mortality for patients. Thoral et al^37^ and Luo et al^46^ showed with calibration curves that their predicted probabilities are very similar to observed probabilities, which indicates that the models are well calibrated.

#### Prediction of complications

Looking at the studies that predicted sepsis (Table 6), Park et al achieved very high AUROC of 0.960 (recurrent neural network) when predicting bacteremia, which is a form of sepsis.^32^ Misra et al^28^ also achieved a high performance (AUROC = 0.948, random forest) when predicting septic shock. Based on sensitivity, Park et al also had the highest performance (Sens = 0.940),^32^ and Misra et al^28^ had the highest specificity of 0.796.

The studies developing models to predict some other complications achieved considerably high AUROC, sensitivity, and specificity (Table 7). Interestingly, Meyer et al, when predicting renal complications, achieved very high accuracy (0.900, recurrent neural network), AUROC (0.960), sensitivity (0.940), specificity (0.860), and PPV and NPV (0.870 and 0.940, respectively). However, as explained previously, Meyer et al^75^ used a balanced dataset for both training and testing data, meaning their model performance is not necessarily reflective of the real-world situation. In their patient demographics, mortality was present in 6.2% of patients, bleeding in 4.9% and renal failure in 1.0%. These proportions show highly imbalanced data, meaning that the models tested on a set where 50% of the patients experienced renal failure reach AUROC of 0.960 is not applicable in a real-world situation where renal failure occurs in only 1% of patients.

Interestingly, other performance measures indicating diagnostic accuracy, such as PPV and NPV were reported by only 5 studies.^22,27,30,41,44^ These 2 performance measures are important to know as these indicate the reliability of the model based on the probability of patient having the outcome predicted by the model.

None of the models that predicted complications reported calibration.

## Discussion

In this scoping review, we have identified and synthesized key information of studies describing the development of dynamic prediction models of patient outcomes in ICU. The studies were analyzed based on the outcomes they predicted, the methods they used to develop the prediction models, and the performance their models achieved. Dealing with missing data in ICUs—as an incredibly data-rich environment—is inevitable,^76^ and therefore an in-depth discussion regarding the handling of missing data and the imbalanced classification problem can be found from the Supplemental Material.

### Predicted Outcomes

By far, the most predicted outcome by studies included in this review was ICU mortality. There are various reasons why predicting mortality is so common.

Firstly, mortality is very straight-forward to define, and is a binary outcome: “dead” or “alive”. Having a clearly defined binary outcome is easier to predict as opposed to more complex multilevel outcomes that have varying levels of definition (eg, such as predicting morbidity, for example). Secondly, mortality is obviously the first and foremost outcome that should be avoided. Thirdly, historically, mortality has always been the main benchmark in the first instance to audit and measure the performance of surgical and medical care.^77^

However, as ICU mortality rates are decreasing^78^ other care quality benchmarks are becoming more important, such as complications.^79^ With an ageing population, morbidity, on the other hand, is becoming more prevalent and is the reason why healthcare systems around the world are struggling to sustain their current “reactive” models of care.^80,81^

The definition of the predicted outcome can be what makes or breaks a prediction model: Because the definition of mortality is clear, there is no bias in the recorded outcome. However, as seen in the studies predicting sepsis, the studies had various definitions. These definitions included internationally approved definitions and classifications of sepsis, such as SIRS, Sepsis-2 and Sepsis-3, however, these agreed-upon definitions and classifications are not perfect,^61^ and are constantly evolving.^14^ Even though sepsis is a widely researched complication, as evidenced by the large number of studies predicting sepsis-related complications in this review, sepsis patients are still often identified too late.^82^ The problem of varying definitions of sepsis outcomes might also explain the lack of prediction of ICU complications in general. For example, acute kidney injury is a relatively common complication,^83^ and is now easily identified based on laboratory measurements using the KDIGO criteria,^70^ which hopefully enables the development of more prediction models for this complication.

Even though electronic health records have come a long way, databases still do not take into account the current consensus definitions of various complications, such as acute kidney injury, sepsis, or the definition of complications in general, which lead to the prediction models being unusable in practice.^84^ Both the sepsis and kidney disease criteria can be calculated once necessary laboratory measurements are taken. This is also the case for other complications that have agreed criteria for diagnosis, such as liver failure.^85^ This means that the time of the onset of the predicted complication can be compromised and shows that further effort in defining complications to enable timely and accurate diagnosis for these outcomes is required.

### Classification Methods and Prediction Frequency

The most common classification method to predict clinical outcomes was logistic regression. This is not surprising as logistic regression has been shown to have very competitive performance compared to more complex machine learning methods.^86–88^ Furthermore, logistic regression is a highly interpretable model, showing which variables are associated with the predicted outcome with easily interpretable odds ratios. Understanding why a prediction model predicts a certain level of probability for a patient to have an outcome is important in practice, so that clinicians know which factors need to be paid attention to.

However, since the studies presented in this review were faced with time-series data, preprocessing methods to capture the temporal aspects of data were required. While many studies summarized the entries for each chosen time window to build their models, the methods handling this type of data varied. Depending on the pre-processing method chosen, this action could introduce further assumptions to the prediction model, which can subsequently make the model less applicable in practice.^89^

Most of the studies predicted outcomes at a certain frequency. Even though all studies in this review developed dynamic, “real-time” models, in reality, the outcomes were predicted less frequently than on a real-time basis. The reason for this is simple: when vital signs are collected very often (eg, every few minutes),^38^ then laboratory results are collected less frequently. Some laboratory results could be collected every few hours, and some daily.^29,33^ This makes a fully real-time prediction impossible.

Often when predicting the outcome every time when new information is entered into the system, not all variables are updated, which means that in reality the variable values with no new information were carried forward from the previous timestamp, as done by a number of studies in this review. As stated by Haukoos et al,^90^ this assumes that the patient state in terms of the carried forward variable stays the same, while in reality this might not be the case.

### MIMIC Databases

The MIMIC databases were commonly used in studies included in this review. While using publicly available databases to develop clinical prediction models helps with the transparency and reproducibility of the models,^91^ there are a few limitations to using certain MIMIC databases. Namely, a third of the studies used the MIMIC-II database, which includes ICU patients’ data collected between 2001 and 2008.^92^ Even though this database was the only 1 available during the time when 9 of the studies were published, for 2 studies, the newer version—MIMIC-III—was already available for almost 2 years.^17,48^

The MIMIC-III database was first released in 2015 and includes ICU patients’ data collected between 2001 and 2012.^54^ MIMIC-IV database was first released in 2020 and includes patient data collected between 2008 and 2019. It also includes clinical data prior to ICU admission.^93^

Understandably, there was a substantial gap between the release of MIMIC-III and MIMIC-IV, and hence many studies were using data that were up to a decade old (eg, Yijing et al's study was published in 2022 and used MIMIC-III^42^). This is a limitation to these studies as the patient population is ever-changing,^78,94^ and clinical interventions, practice and policies change constantly.^95^ In addition, with more studies investigating electronic health records, the data quality in clinical systems is improving.^96^ Hence, using a data that was recorded many years ago might make the developed clinical prediction models not usable in current patient population.

An alternative database to the MIMIC is the eICU database, released in 2018. The eICU database includes ICU data collected between 2014 and 2015.^55^ Even though the dataset is newer, only 3 studies used this dataset.^33,43,46^ The lack of usage of eICU might be that the MIMIC databases have been widely used in the literature for over a decade, whereas the eICU has been available for 5 years only.

Another limitation of using the MIMIC and eICU is that they are both US-based databases. Although, eICU consists of data from 208 US hospitals, the MIMIC databases consist of patient data only from the Beth Israel Deaconess Medical Center. Even though a third of the studies were based in the USA and used the MIMIC databases, 12 studies were conducted outside of the USA and still used the MIMIC databases. This means that the majority (23 out of 36) of the studies have developed US-centric prediction models which might not necessarily be applicable in other countries, or even within the general US patient population.

Overall, the availability of large open-source ICU databases brings a lot of opportunities for clinical data analytics innovation. These databases are great sandpits to test and develop new methodologies and approaches to improve clinical outcomes.^91^ However, to be able to apply models in practice, more recent and diverse data should be used to ensure the applicability of the models in a current, up-to-date patient population.

### Clinical Implications, Implementation, and Adoption of Prediction Models

While there are numerous prediction models in medicine, the information about the implementation and adoption of these prediction models is limited. Among the studies included in this review, none have been reported to be applied in clinical practice. An important measure of model's applicability in a specific patient population is calibration.^97^ In this review, only 5 studies reported measuring calibration of their prediction models,^19,25,34,37,46^ which indicates low reporting standards of currently developed prediction models, as also evidenced by our findings and discussion about the missing data and data imbalance approaches in Supplemental Material.

As stated by Seneviratne et al^98^: “Very few of these algorithms ever make it to the bedside; and even the most technology-literate academic medical centers are not routinely using AI in clinical workflows”*.* While prediction models in healthcare have made some major progress in deployment and medical image interpretation, implementation, accountability and ethics still remain a challenge.^99^ The main factors that influence the successful implementation of prediction models are perceived ease of use or usefulness, performance or effort expectancy, and social influence.^100^ To enable more wide-spread implementation of current prediction models, shifting the focus from optimizing performance metrics to practical aspects of model design, such as actionability, safety and utility, and consulting the potential users of the model could be useful.^98^

While a considerable amount of effort is still required to develop usable, fit-for-purpose clinical prediction models, the implications for clinical practice can be extremely useful. Being able to identify patients who are at high risk for a particular outcome, such as disease progression or a postoperative complication, would allow directing interventions, such as more intensive monitoring or treatment, to those who are most likely benefit. Consequently, these prediction models would help to improve the quality of care and patient outcomes.

## Conclusion

This review analyzed published studies that predicted patient outcomes in critical care in a dynamic manner. The studies included show that there is a strong interest in developing dynamic prediction models for various patient outcomes, however, the models developed so far have limitations. Most studies narrowly focus on mortality when there is a range of other serious, but well-defined, complications, such as acute kidney injury that would also benefit from further investigation. Furthermore, there is often a lack of sufficient details included across studies, specifically on how missing data were handled in the predictive models’ development. Finally, more emphasis should be placed on testing the models in local databases that are appropriate for the potential demographic which the prediction model is intended for. Prediction models have an enormous potential to aid in decision-making and diagnostics in critical care setting, where the amount of data is vast. Therefore, more emphasis should be placed on predicting complications, and carrying out validation and evaluation studies to allow for the successful implementation of the models.

## Supplemental Material

sj-docx-1-jic-10.1177_08850666231166349 - Supplemental material for Dynamic Prediction of Patient Outcomes in the Intensive Care Unit: A Scoping Review of the State-of-the-Art Click here for additional data file. Supplemental material, sj-docx-1-jic-10.1177_08850666231166349 for Dynamic Prediction of Patient Outcomes in the Intensive Care Unit: A Scoping Review of the State-of-the-Art by Linda Lapp, Marc Roper, Kimberley Kavanagh, Matt-Mouley Bouamrane and Stefan Schraag in Journal of Intensive Care Medicine
