# Supplementary material for: Dynamic Prediction of Patient Outcomes in the Intensive Care Unit: A Scoping Review of the State-of-the-Art
Source: J Intensive Care Med. 2023 Apr 5;38(7):575–91. doi: 10.1177/08850666231166349 (PMC10302367; doi:10.1177/08850666231166349)
Supplement: sj-docx-1-jic-10.1177_08850666231166349 - Supplemental material for Dynamic Prediction of Patient Outcomes in the Intensive Care Unit: A Scoping Review of the State-of-the-Art [file sj-docx-1-jic-10.1177_08850666231166349.docx]

# Supplementary File

# Summary and Discussion on Approaches to Missing Data

## Methods Used by Studies

Dealing with missing data in ICUs − as an incredibly data-rich environment − is inevitable^1^. The reasons for missing data in medical records, including ICU records, are complex: data can be missing randomly due to errors in automatic recording devices or due to human error^2^, however, data can also be missing because certain tests are done at certain intervals or only if needed. A large number of missing values in medical databases represents a challenge as a patient’s health state needs to be examined even when no observations are available^3^, and hence using various approaches to treat missing data can be beneficial when dealing with these errors. Table S1 shows how missing data were handled by studies included in this review.

**Table S1.** Missing data approaches used by each included study.

| Missing Data Approach | Number of Studies | Author and Year | Amount of missing data |
| --- | --- | --- | --- |
| Imputation | 11 | Caballero 2015 | 34% estimated |
|  |  | Dummitt 2018 | 0-94.7% |
|  |  | Hu 2022 | Not reported |
|  |  | Lehman 2015 | Not reported |
|  |  | Ma 2019 | 0-93.21% |
|  |  | Misra 2021 | Not reported |
|  |  | Park 2020 | Not reported |
|  |  | Pattalung 2021 | Not reported |
|  |  | Ryan 2022 | 5.7% - 96.0% |
|  |  | Shashikumar 2017 | Not reported |
|  |  | Zhao 2021 | Not reported |
| Carrying Forward/Interpolation | 8 | Dummitt 2018 | 0-94.7% |
|  |  | Hu 2022 | Not reported |
|  |  | Joshi 2012 | Not reported |
|  |  | Meyer 2018 | Not reported |
|  |  | Park 2020 | Not reported |
|  |  | Pattalung 2021 | Not reported |
|  |  | Yijing 2022 | Not reported |
|  |  | Xia 2019 | Not reported |
| Informative Missingness | 2 | Deasy 2020 | Not reported |
|  |  | Huddar 2016 | Not reported |
| Removal of variables | 3 | Dummitt 2018 | 0-94.7% |
|  |  | Feng 2021 | Not reported |
|  |  | Misra 20021 | Not reported |
| Removal of entries | 3 | Lehman 2015 | Not reported |
|  |  | Raj 2019 | Mean = 70 values for intracranial pressure, 78 values for mean arterial pressure and 70 for cerebral perfusion pressure |
|  |  | Silva 2006 | 4 entries |
| Model "handles" | 2 | Zhao 2021 | Not reported |
|  |  | Luo 2022 | 0%-52.28% |
| Unclear | 4 | Bhattacharya 2018 | Not reported |
|  |  | Gultepe 2014 | Not reported |
|  |  | Thoral 2021 | Not reported |
|  |  | Yee 2019 | Not reported |
| None reported | 11 | Ghosh 2017 | Not reported |
|  |  | Henry 2015 | Not reported |
|  |  | Hernandez 2021 | Not reported |
|  |  | Hug 2009 | Not reported |
|  |  | Johnson 2017 | Not reported |
|  |  | Lee 2010 | Not reported |
|  |  | Lehman 2013 | Not reported |
|  |  | Mao 2012 | Not reported |
|  |  | Mohammed 2020 | Not reported |
|  |  | Nemati 2018 | Not reported |
|  |  | van Wyk 2019 | Not reported |

### Imputation Methods

Imputation methods are used to fill in missing values with another, probable value. Using imputation methods can be beneficial as this allows including patients who can have relevant features for analysis but could be otherwise be excluded from analysis due to data collection or recording errors^4^. Imputation methods were used by almost a third of the studies (n=9 studies).

Shashikumar et al.^5^, Park et al.^6^, Hu et al.^7^ and Ryan et al.^8^ used mean imputation to replace missing values. Mean imputation is a single imputation method where the missing values are replaced by the mean of the observed values of the variable with missing data^4^. Even though mean imputation is a very straight-forward method, it can cause severely biased estimates due to changing the variance of the data^9^. That being said, if the missingness of data is low (<10%) and the variables with missing values are not highly correlated with the predicted outcome, the effects on the reliability of predicted outcome are marginal^10^.

Dummitt et al.^11^ and Zhao et al.^12^ used the population median to populate missing values. This was also done for clear outliers that were removed from dataset and replaced with new values based on the population median^11^. Median imputation is very similar to mean imputation, but instead of replacing the missing values with the population mean, the median is used. Similar to mean imputation, median imputation is also a very straight-forward approach, however, takes into account that real-life data are not always normally distributed^13^. It has been shown to perform similarly well as more sophisticated imputation methods^14^.

Interestingly, Pattalung et al. replaced the missing values with the value “-1”^15^. In their study they do not explain further why this decision was made and what assumptions they had when undertaking this approach. In practice, replacing missing values with a certain agreed-upon value would simplify the usage of a prediction model if a clinician was faced with missing values. However, this can significantly alter the probability for a patient to have the predicted outcome. Pattalung et al.’s study reports that their model had slight differences in the variable importance values when trained on two different databases^15^. This result can be due to differences in completeness of data in the datasets, however, the authors did not report the percentage of missing data in their study.

Caballero et al. used Regularised Expectation Maximization (EM) to fill out the missing values^3^. EM is based on iterated analyses of linear regressions of variables with missing values on variables with available values, with regression coefficients estimated by ridge regression, a regularised regression method in which a continuous regularisation parameter controls the filtering of the noise in the data. The regularisation parameter is determined by generalised cross-validation, such as to minimise, approximately, the expected mean-squared error of the imputed values. The regularised EM algorithm has been shown to be able to estimate missing values for various types of missing data problems^16^.

Lehman et al. used Gaussian noise imputation for time-series data to replace missing or invalid values in their dataset^17^. There is a wealth of information about Gaussian processes handling missing values in the literature, however Lehman et al. used logistic regression (see Section 3.3.3) for their prediction model. Since they did not provide a reference to what exactly they mean by using Gaussian noise to “fill in the missing or invalid values”^17^, it is unclear what processes they carried out to handle missing data.

Ma et al. used a tree-based estimation algorithm to replace missing values^18^. To do that, they replaced the missing values with the value “-1000” as this value was very different from the non-missing values. This was done for the estimation algorithm to treat these missing values differently. However, Ma et al. do not provide a reference to what kind of tree-based estimation algorithm they used for the imputation^18^. There are various tree-based algorithms that have been developed to impute missing data, including methods developed by D’Ambrosio et al.^19^, Vateekul et al.^20^, and Rahman et al.^21^. Not specifying which tree-based method was used for missing data imputation makes the developed models not reproducible and reduces the chance for the models to be implemented in clinical practice due to lack of transparency^22^.

Misra et al. used random forest imputation to replace missing values in their data^23^. Random forest imputation, i.e., missForest is a pattern-based method, which can be applied to any kind of data (numerical or categorical). It requires no tuning of parameters or assumptions about the data distribution^24^. MissForest has been shown to outperform most other methods of missing data imputation, showing low imputation error and maintaining predictive ability in clinical prediction models^25,26^. However, as per Sperrin et al., while using multiple imputation methods is common in model development and validation, it may overestimate the performance of deployed prediction models due to multiple imputation methods often requiring outcome to inform imputation, which is, however, unavailable at the time of prediction^27^.

### Carrying Forward and Interpolation

Six studies used the “carrying forward” method, where the patients’ most recent reading from earlier in the database is used, if available. This method was used by Dummitt et al.^11^, Joshi et al.^28^, Meyer et al.^29^, Park et al.^6^, Pattalung et al.^15^, and Yijing et al.^30^.

An alternative to the carrying forward method is linear interpolation, used by Xia et al.^31^. In one-dimensional data sequence, linear interpolation estimates the missing value based on the two data points adjacent to the points that has a missing value^32^. Therein lies the difference between interpolation and carrying forward: for carrying forward, the missing values are replaced with the previously recorded value; for interpolation, however, the missing values are replaced with a value that has been calculated based on also the next available value. The primary assumption of carrying forward method is that the value did not change from the previously recorded value, which can be clinically justified, depending on the frequency of the measurement and how often the model updates. The interpolation method can be more reflective of the changes in patient’s health as it takes into account the next recorded value but also makes an assumption that the trajectory between the two data points is linear^32^.

### Removal Methods

Three studies excluded variables from the analysis that had a high level of missing data. Dummitt et al., for example, removed variables with >89% of missing data, however kept other variables in the analysis that were deemed essential for their prediction task^11^. Feng et al. and Misra et al. removed variables with missing rate over 40%^23,33^.

Three studies also excluded records with a high number of missing values. Silva et al. removed entries due to missing values, however the total number of remaining records was very high (13,164 records)^34^. Lehman et al. excluded patients with more than 15% of missing or invalid samples^17^. Raj et al., however, excluded the patient only if the missing values fell in a specific time window^35^.

### Informative Missingness

Two studies used informative missingness to approach missing data^3637^. Deasy et al., for example, explained that the missing data included in their recurrent neural networks model was treated as separate discrete events where models used these as “informative missingness”^36^. Informative missingness, as Huddar et al. argue, means that the data are missing because clinicians deemed the test unnecessary. This provides information on the patient’s status by showing that the patient was too healthy to need a test or receive medication. The missingness can be incorporated, for example, by creating a separate category for a variable that has missing values^37^.

Incorporating informative missingness needs to be decided upon based on how the developed prediction model will be used in practice. If a system is incorporated in an electronic health system, and calculates probabilities for predicted outcomes automatically, the effect of informative missingness can be hidden from the clinician. However, if risk prediction is done by hand using a scoring system, the clinician is able to make an informed decision by also including informative missingness^37^. Since Deasy et al. developed a prediction model that incorporates all data from the electronic health record^36^, and Huddar et al. developed a model that is incorporated with the electronic health record^38^, it is difficult to know how much the developed models actually take this informative missingness into account.

### Model-Handled Methods

Zhao et al.^12^ and Luo et al.^39^ explain that the boosting machine learning methods they were using in their analysis can use missing data when making the prediction. These models were CatBoost, light Gradient Boosting, Gradient Boosting Machine (GBM) for Zhao et al.’s study, and Extreme Gradient Boosting (XGBoost) for both studies.

Even though Zhao et al. stated that no entries with missing data were removed from analysis, they did not report what the rate of missingness in the data was. Conversely, Luo et al. reported the missingness to be up to 52.28% for certain variables. It has been shown that the amount and the distribution of missing data plays an important role when developing predictive models in terms of variability and bias of the results^9,10,40^.

### Other Approaches

Four studies did not explain their methods for handling missing data very clearly. Bhattacharya et al. mentioned that data were “cleaned”, but no further information was given^41^.

Gultepe et al. stated that no missing data were included in the study, meaning that assumably only records with complete data were included in the study^42^.

Thoral et al. mentioned that values with no biological plausibility were removed from analysis, however, did not report what was done with these missing values in the data as they did not mention removing variables with these values or patient records with these values^43^.

Yee et al. reported interpreting missing values as “not measured”, but they did not explain how these “not measured” values were handled in their analysis^44^.

In total, eleven studies did not report any action taken regarding missing values^45–55^.

## Discussion

The fact that a third of the included studies did not report how missing data were handled in their research is surprising. This is a clear limitation of these studies as missing data in electronic health records is highly prevalent^1^. Reporting how missing data were handled when developing a clinical prediction model is a critical step for transparency, as also required by the transparent reporting of a multivariable prediction model for individual prognosis or diagnosis (TRIPOD) reporting guidance^22^.

As stated by Tsvetanova et al., currently there is no clear guidance on how to handle missing data when developing, validating and implementing clinical prediction models^56^. As seen in this review, various imputation methods, specifically mean and median imputation, are very popular ways of handling missing data. Single imputation methods, such as mean and median imputation, and even replacing missing values with a specific value, like Pattalung et al. did, can introduce some bias to the results. However, alternative methods, such as missForest imputation and k-nearest neighbours imputation, are more computationally intensive and therefore potentially incompatible when producing rapid, deployable prediction models for serious complications, like septic shock, in real-time^10,11,56^. In general, the description of methods used to handle missing data were not adequately reported in the included studies.

The rate of missing data in studies was also not very well documented. The TRIPOD adherence assessment requires transparency on (1) whether there is missing data, (2) the method for handling missing data, (3) details of the software used to handle missing data, and (4) description of which variables were affected by the missing data methods^22^. Only five studies stated clearly how much missing data there was in their datasets^3,11,18,34,35^. This means that for most studies, how many variables or patient records were removed from their data is unknown, how much of the data were replaced with imputation methods, carry-forward or interpolation methods, and how much data had to be handled by the prediction models. Often the rate of missingness in development cohort is different from the cohort the prediction model is deployed on^27^. Hence, not reporting the missingness is a serious limitation to the studies in general as it reduces the transparency of the model development, and hence makes it less clear how applicable the models are to use in practice^22^.

Finally, only two studies used models that are robust enough to handle missing data^12,39^. There are now various methods available that have been developed to take missing data into account, including CatBoost^12^, C5.0^57^ and BARTm^58^.

# Summary and Discussion on Approaches to the Imbalanced Classification Problem

## Balancing Approaches

While it has been shown that imbalance correction can lead to models presenting strong miscalibration and therefore reduce clinical utility of the models due to inaccurate probability estimates^59^, in total, n=15 studies mentioned facing an imbalanced classification problem in their analysis and reported how they approached this problem (Table S2).

**Table S2.** Approaches for imbalanced classification problem used by studies.

| Imbalanced Classification | Number of Studies | Author and Year |
| --- | --- | --- |
| Balanced by method | 7 | Huddar 2016 |
|  |  | Lee 2010 |
|  |  | Mao 2012 |
|  |  | Misra 2021 |
|  |  | Mohammed 2020 |
|  |  | Pattalung 2021 |
|  |  | Silva 2006 |
| Performance Measures | 4 | Johnson 2017 |
|  |  | Ma 2019 |
|  |  | Ryan 2022 |
|  |  | Thoral 2021 |
| Data selection | 2 | Meyer 2018 |
|  |  | van Wyk 2019 |
| Modelling method approach | 2 | Caballero 2015 |
|  |  | Dummitt 2018 |

### Balanced by Method

Seven studies reported using a specific method to manipulate the sample to achieve a balanced dataset. Two studies used Synthetic Minority Oversampling Technique (SMOTE) on their training sets^23,38^. Misra et al. also reported using upsampling^60^, however, it is unclear why both SMOTE^61^ and upsampling were used, and how these two methods were used at the same time^23^. Over-sampling was also used by Pattalung et al. on their training data^15^.

Lee et al. reported using subsampling^62^ in their training data to achieve a balanced data set^51^. Undersampling^63^ was used by Mao et al.^53^, and Silva et al.^34^. Mohammed et al. used a Bayesian bootstrap method^64^ to balance controls with cases^54^.

Most studies carried out these sampling methods on training sets only, and left the testing sets as original, as is recommended^65^, however, Mao et al.^53^ and Mohammed et al.^54^ carried out the balancing methods on both training and testing data. This approach is not recommended^65^ as this means that their models were tested on balanced datasets which do not reflect the proportion of cases and controls as it does in practice. In addition, this means that the predicted probabilities can be incorrect and not be applied in a real-world situation^59^.

### Data Selection

Two studies approached the imbalanced classification problem by sampling their data as equally sized case and control groups^29,47^. This means that both of these studies worked with balanced training and testing datasets, which makes the predicted probabilities by models not applicable in a real-world setting^59^.

### Choosing Appropriate Modelling Method

Caballero et al.^3^ and Dummitt et al.^11^ approached the imbalanced classification problem by choosing predictive modelling methods that have been shown to be robust when handling imbalanced datasets. Caballero et al. report that the naïve Bayes classifier they were using for text classification had shown good predictive performance for unbalanced classes^3^. Dummitt et al. checked that the number of events per variable was kept above the recommended thresholds for the classification methods they used to ensure the model coefficients would not be biased by the case balance^11^. It was, however, not explained how this was achieved.

### Choosing Appropriate Performance Measures

Four studies reported that due to the imbalanced classification problem, instead of reporting only the accuracy of the model, they also reported area under the receiver operating characteristic curve (AUROC) and/or area under the precision-recall curve (AUPRC)^8,18,43,50^. These performance measures have been shown to give a better reflection of how the model recognises both positive and negative classes^66,67^. However, it is worth noting that the prevalence of the predicted outcome for Ryan et al.’s study was 50%, which is surprisingly high for the investigated patient population and might indicate that the data collection was carried out, having a balanced approach in mind. This, however was not reported in their paper.

## Discussion

A third of the studies were dealing with highly imbalanced classification problems, where the prevalence of the predicted outcome was <10%. The four main methods identified for approaching the class imbalance: (1) using balancing methods, (2) selecting equal case and control groups, (3) using performance measures reflective of class imbalance, and (4) using modelling methods shown to perform well with imbalanced outcome.

There were some limitations in the included studies in terms of reporting imbalanced classification challenges. Surprisingly, four studies^3,34,49,50^ did not report the prevalence of the outcome they were predicting. Knowing the prevalence and the methods to pre-process the data (e.g., missing data and balancing methods) helps to understand how applicable the models are in clinical practice with the real-world data. In addition, when most studies applied the balancing methods correctly, the studies by Mao et al.^53^ and Mohammed et al.^54^ applied the balancing method on the full dataset, before partitioning the training and testing dataset. This is not a recommended approach^65^. In addition, the study by Meyer et al.^29^ and van Wyk et al.^47^ chose equal case and control cohorts for the full dataset, again, before dividing the data into training and testing dataset. For these four studies, this means that their models were evaluated on the testing dataset that was balanced, which does not reflect the real-world data, and leas to poor calibration and reduced utility in clinical setting^59^.

In general, it is surprising that so many studies used balancing methods to solve the imbalanced classification problem, especially, if so many predictive modelling methods, including common ones, like logistic regression and random forest, have been shown to handle imbalanced classes well^68–70^.

It is known that balancing methods or developing models on training sets that have a balanced outcome can lead to poor calibration, where the probability of the predicted outcome is overestimated. As said by van den Goorbergh et al., *“Outcome imbalance is not a problem in itself”* and *“imbalance correction may even worsen model performance”*^59^. This can also explain that Meyer et al.’s results of AUROC of 0.95, 0.87 and 0.96 for mortality, bleeding and kidney failure, respectively, may be an overestimation of how well the outcomes were predicted, especially because in their original data, the prevalence of these complications was 6.2% for mortality, 4.9% for bleeding and 1.0% for renal failure.

The models by Mao et al., Mohammed et al., and van Wyk et al. achieved only moderate performance measures. This is surprising as they tested their models on balanced datasets.

# Supplementary File References

1. Mazzali C, Duca P. Use of administrative data in healthcare research. *Intern Emerg Med*. 2015;10:517-524. doi:10.1007/s11739-015-1213-9

2. Goldberg SI, Niemierko A, Turchin A. Analysis of data errors in clinical research databases. *AMIA Annu Symp Proc*. Published online 2008:242-246.

3. Caballero K, Akella R. Dynamically modeling Patient’s health state from electronic medical records: A time series approach. *Proc ACM SIGKDD Int Conf Knowl Discov Data Min*. 2015;2015-Augus:69-78. doi:10.1145/2783258.2783289

4. Jamshidian M, Mata M. Advances in Analysis of Mean and Covariance Structure when Data are Incomplete. In: *Handbook of Latent Variable and Related Models*. ; 2007:21-44. doi:10.1016/B978-044452044-9/50005-7

5. Shashikumar SP, Stanley MD, Sadiq I, et al. Early sepsis detection in critical care patients using multiscale blood pressure and heart rate dynamics. *J Electrocardiol*. 2017;50(6):739-743. doi:10.1016/j.jelectrocard.2017.08.013

6. Park HJ, Jung DY, Ji W, Choi CM. Detection of bacteremia in surgical in-patients using recurrent neural network based on time series records: development and validation study. *J Med Internet Res*. 2020;22(8):e19512. doi:10.2196/19512

7. Hu J, Kang XH, Xu FF, Huang KZ, Du B, Weng L. Dynamic prediction of life-threatening events for patients in intensive care unit. *BMC Med Inform Decis Mak*. 2022;22(276). doi:10.1186/s12911-022-02026-x

8. Ryan CT, Zeng Z, Chatterjee S, et al. Machine learning for dynamic and early prediction of acute kidney injury after cardiac surgery. *J Thorac Cardiovasc Surg*. Published online 2022. doi:10.1016/j.jtcvs.2022.09.045

9. Jamshidian M, Bentler PM. ML estimation of mean and covariance structures with missing data using complete data routines. *J Educ Behav Stat*. 1999;24(1):21-41. doi:https://doi.org/10.2307/1165260

10. Tsikriktsis N. A review of techniques for treating missing data in OM survey research. *J Oper Manag*. 2005;24(1):53-62. doi:https://doi.org/10.1016/j.jom.2005.03.001

11. Dummitt B, Zeringue A, Palagiri A, Veremakis C, Burch B, Yount B. Using survival analysis to predict septic shock onset in ICU patients. *J Crit Care*. 2018;48:339-344. doi:10.1016/j.jcrc.2018.08.041

12. Zhao QY, Liu LP, Luo JC, et al. A machine-learning approach for dynamic prediction of sepsis-induced coagulopathy in critically ill patients with sepsis. *Front Med*. 2021;7(637434). doi:10.3389/fmed.2020.637434

13. Jadhav A, Pramod D, Ramanathan K. Comparison of performance of data imputation methods for numeric dataset. *Appl Artif Intell*. 2019;33(10):913-933. doi:10.1080/08839514.2019.1637138

14. Berkelmans GFN, Read SH, Gudbjörnsdottir S, et al. Population median imputation was noninferior to complex approaches for imputing missing values in cardiovascular prediction models in clinical practice. *J Clin Epidemiol*. 2022;145:70-80. doi:10.1016/j.jclinepi.2022.01.011

15. Pattalung TN, Ingviya T, Chaichulee S. Feature explanations in recurrent neural networks for predicting risk of mortality in intensive care patients. *J Pers Med*. 2021;11(934). doi:https://doi.org/10.3390/jpm11090934

16. Schneider T. Analysis of Incomplete Climate Data: Estimation of Mean Values and Covariance Matrices and Imputation of Missing Values. *J Clim*. 2001;14(5):853-571. doi:https://doi.org/10.1175/1520-0442(2001)014<0853:AOICDE>2.0.CO;2

17. Lehman LWH, Adams RP, Mayaud L, et al. A Physiological Time Series Dynamics-Based Approach to Patient Monitoring and Outcome Prediction. *IEEE J Biomed Heal Informatics*. 2015;19(3):1068-1076. doi:10.1109/JBHI.2014.2330827.A

18. Ma J, Lee DKK, Perkins ME, Pisani MA, Pinker E. Using the shapes of clinical data trajectories to predict mortality in ICUs. *Crit Care Explor*. 2019;1(e0010). doi:10.1097/CCE.0000000000000010

19. D’Ambrosio A, Aria M, Siciliano R. Accurate Tree-based Missing Data Imputation and Data Fusion within the Statistical Learning Paradigm. *J Classif*. 2012;29:227-258. doi:https://doi.org/10.1007/s00357-012-9108-1

20. Vateekul P, Sarinnapakorn K. Tree-Based Approach to Missing Data Imputation. In: *2009 IEEE International Conference on Data Mining Workshops*. ; 2009:70-75. doi:10.1109/ICDMW.2009.92

21. Rahman G, Islam Z. A Decision Tree-based Missing Value Imputation Technique for Data Pre-processing. In: *Proceedings of the 9-Th Australasian Data Mining Conference*. ; 2011:41-50.

22. Moons KGM, Altman DG, Reitsma JB, et al. Transparent reporting of a multivariable prediction model for individual prognosis or diagnosis (TRIPOD): Explanation and elaboration. *Ann Intern Med*. 2015;162(1):W1-73. doi:10.7326/M14-0698

23. Misra D, Avula V, Wolk DM, et al. Early detection of septic shock onset using interpretable machine learners. *J Clin Med*. 2021;10(301). doi:10.3390/jcm10020301

24. Stekhoven DJ, Bühlmann P. MissForest - non-parametric missing value imputation for mixed-type data. *Bioinformatics*. 2012;28(1):112-118. doi:https://doi.org/10.1093/bioinformatics/btr597

25. Shah AD, Bartlett JW, Carpenter J, Nicholas O, Hemingway H. Comparison of random forest and parametric imputation models for imputing missing data using MICE: a caliber study. *Am J Epidemiol*. 2014;179(6):764-774. doi:10.1093/aje/kwt312

26. Waljee AK, Mukherjee A, Singal AG, et al. Comparison of imputation methods for missing laboratory data in medicine. *BMJ Open*. 2013;3(e002847). doi:http://dx.doi.org/10.1136/bmjopen-2013-002847

27. Sperrin M, Martin GP, Sisk R, Peek N. Missing data should be handled differently for prediction than for description or causal explanation. *J Clin Epidemiol*. 2020;125:183-187. doi:https://doi.org/10.1016/j.jclinepi.2020.03.028

28. Joshi R, Szolovits P. Prognostic physiology: modeling patient severity in Intensive Care Units using radial domain folding. In: *AMIA Annual Symposium Proceedings*. ; 2012:1276-1283.

29. Meyer A, Zverinski D, Pfahringer B, et al. Machine learning for real-time prediction of complications in critical care: a retrospective study. *Lancet Respir Med*. 2018;6(12):905-914. doi:10.1016/S2213-2600(18)30300-X

30. Yijing L, Wenyu Y, Kang Y, et al. Prediction of cardiac arrest in critically ill patients based on bedside vital signs monitoring. *Comput Methods Programs Biomed*. 2022;214(106568). doi:https://doi.org/10.1016/j.cmpb.2021.106568

31. Xia J, Pan S, Zhu M, et al. A long short-term memory ensemble approach for improving the outcome prediction in intensive care unit. *Comput Math Methods Med*. 2019;2019. doi:https://doi.org/10.1155/2019/8152713

32. Huang G. Missing data filling method based on linear interpolation and lightgbm. *J Phys Conf Ser*. 2021;1754(012187).

33. Feng X, Pan S, Yan M, et al. Dynamic prediction of late noninvasive ventilation failure in intensive care unit using a time adaptive machine model. *Comput Methods Programs Biomed*. 2021;208(106290). doi:https://doi.org/10.1016/j.cmpb.2021.106290

34. Silva A, Cortez P, Santos M., Gomes L, Neves J. Mortality assessment in intensive care units via adverse events using artificial neural networks. *Artif Intell Med*. 2006;36(3):223-234. doi:10.1016/j.artmed.2005.07.006

35. Raj R, Luostarinen T, Pursiainen E, et al. Machine learning-based dynamic mortality prediction after traumatic brain injury. *Sci Rep*. 2019;9(1):17672. doi:https://doi.org/10.1038/s41598-019-53889-6

36. Deasy J, Lio P, Ercole A. Dynamic survival prediction in intensive care units from heterogeneous time series without the need for variable selection or curation. *Nature*. 2020;10(22129). doi:https://doi.org/10.1038/s41598-020-79142-z

37. Groenwold RHH. Informative missingness in electronic health record systems: the curse of knowing. *Diagnostic Progn Res*. 2020;4(8). doi:https://doi.org/10.1186/s41512-020-00077-0

38. Huddar V, Desiraju BK, Rajan V, Bhattacharya S, Roy S, Reddy CK. Predicting Complications in Critical Care Using Heterogeneous Clinical Data. *IEEE Access*. 2016;4:7988-8001. doi:10.1109/ACCESS.2016.2618775

39. Luo XQ, Yan P, Duan SB, et al. Development and validation of machine learning models for real-time mortality prediction in critically ill patients with sepsis-associated acute kidney injury. *Front Med*. 2022;9. doi:10.3389/fmed.2022.853102

40. Prevosti FJ, Chemisquy MA. The impact of missing data on real morphological phylogenies: influence of the number and distribution of missing entries. *Cladistics*. 2010;26:326-339. doi:https://doi.org/10.1111/j.1096-0031.2009.00289.x

41. Bhattacharya S, Huddar V, Rajan V, Reddy C. A dual boundary classifier for predicting acute hypotensive episodes in critical care. *PLoS One*. 2018;13(2). doi:10.1371/journal.pone.0193259

42. Gultepe E, Green JP, Nguyen H, Adams J, Albertson T, Tagkopoulos I. From vital signs to clinical outcomes for patients with sepsis: A machine learning basis for a clinical decision support system. *J Am Med Informatics Assoc*. 2014;21(2):315-325. doi:10.1136/amiajnl-2013-001815

43. Thoral PJ, Fornasa M, de Bruin DP, et al. Explainable machine learning on AmsterdamUMCdb for ICU discharge decision support: uniting intensivists and data scientists. *Crit Care Explor*. 2021;3(9). doi:10.1097/CCE.0000000000000529

44. Yee CR, Narain NR, Akmaev VR, Vemulapalli V. A Data-Driven Approach to Predicting Septic Shock in the Intensive Care Unit. *Biomed Inform Insights*. 2019;4(11). doi:10.1177/1178222619885147

45. Ghosh S, Li J, Cao L, Ramamohanarao K. Septic shock prediction for ICU patients via coupled HMM walking on sequential contrast patterns. *J Biomed Inform*. 2017;66:19-31. doi:10.1016/j.jbi.2016.12.010

46. Henry K., Hager D., Pronovost P., Saria S. A targeted real-time early warning score (TREWScore) for septic shock. *Sci Transl Med*. 2015;7(299):299ra122. doi:10.1126/scitranslmed.aab3719

47. van Wyk F, Khojandi A, Mohammed A, Begoli E, Davis RL, Kamaleswaran R. A minimal set of physiomarkers in continuous high frequency data streams predict adult sepsis onset earlier. *Int J Med Inform*. 2019;122:55-62. doi:10.1016/j.ijmedinf.2018.12.002

48. Hernandez L, Kim R, Tokcan N, et al. Multimodal tensor-based method for integrative and continuous patient monitoring during postoperative cardiac care. *Artif Intell Med*. 2021;113(102032). doi:https://doi.org/10.1016/j.artmed.2021.102032

49. Hug CW, Szolovits P. ICU acuity: real-time models versus daily models. *AMIA Annu Symp Proc*. 2009;2009:260-264.

50. Johnson AEW, Mark RG. Real-time mortality prediction in the Intensive Care Unit. *AMIA Annu Symp Proc*. Published online 2017:994-1003.

51. Lee J, Mark RG. An investigation of patterns in hemodynamic data indicative of impending hypotension in intensive care. *Biomed Eng Online*. 2010;9:1-17. doi:10.1186/1475-925X-9-62

52. Lehman LWH, Nemati S, Adams RP, Moody G, Malhotra A, Mark RG. Tracking progression of patient state of health in critical care using inferred shared dynamics in physiological time series. In: *IEEE Engineering in Medicine and Biology Society*. ; 2013:7072-7075. doi:10.1109/EMBC.2013.6611187

53. Mao Y, Chen W, Chen Y, Lu C, Kollef M, Bailey T. An integrated data mining approach to real-time clinical monitoring and deterioration warning. *Proc ACM SIGKDD Int Conf Knowl Discov Data Min*. 2012;(November 2014):1140-1148. doi:10.1145/2339530.2339709

54. Mohammed A, van Wyk F, Chinthala L, Khojandi A. Temporal differential expression of physiomarkers predicts sepsis in critically ill adults. *Shock*. 2020;56(1):58-64. doi:10.1097/shk.0000000000001670

55. Nemati S, Holder A, Razmi F, Stanley MD, Clifford GD, Buchman TG. An Interpretable Machine Learning Model for Accurate Prediction of Sepsis in the ICU. *Crit Care Med*. 2018;46(4):547-553. doi:10.1097/CCM.0000000000002936

56. Tsvetanova A, Sperrin M, Peek N, Buchan I, Hyland S, Martin GP. Missing data was handled inconsistently in UK prediction models: a review of method used. *J Clin Epidemiol*. 2021;140:149-158.

57. Pandya R, Pandya J. C5.0 Algorithm to Improved Decision Tree with Feature Selection and Reduced Error Pruning. *Int J Comput Appl*. 2015;117(16).

58. Kapelner A, Bleich J. Prediction with Missing Data via Bayesian Additive Regression Trees. *arXiv*. Published online 2013.

59. van den Goorbergh R, van Smeden M, Timmerman D, van Calster B. *The Harm of Class Imbalance Corrections for Risk Prediction Models: Illustration and Simulation Using Logistic Regression*.; 2022.

60. Barr JR, Sobel M, Thatcher T. Upsampling, a comparative study with new ideas. In: *2022 IEEE 16th International Conference on Semantic Computing (ICSC)*. ; 2022:318-321.

61. Chawla N V., Bowyer KW, Hall LO, Kegelmeyer WP. SMOTE: Synthetic Minority Oversampling Technique. *J Artif Intell Res*. 2002;16. doi:10.1613jair.953

62. Politis DN. *Subsampling*. Springer Science & Business Media; 1999.

63. Fujiwara K, Huang Y, Hori K, et al. Over- and Under-sampling approach for extremely imbalanced and small minority data problem in health record analysis. *Front Public Heal*. Published online 2020.

64. Rubin DB. The Bayesian Bootstrap. *Ann Stat*. 1981;9(1):130-134.

65. Vandewiele G, Dehaene I, Kovacs G, et al. Overly Optimistic Prediction Results on Imbalanced Data: a Case Study of Flaws and Benefits when Applying Over-sampling. *Artif Intell Med*. 2021;111(1).

66. Gu Q, Zhu L, Cai Z. Evaluation measures of the classification performance of imbalanced data sets. In: *ISICA 2009: Computational Intelligence and Intelligent Systems*. ; 2009:461-471.

67. Wardhani NWS, Rochayani MY, Iriany A, Sulistyono AD, Lestantyo P. Cross-validation Metrics for Evaluating Classification Performance on Imbalanced Data. *2019 Int Conf Comput Control Informatics its Appl*. Published online 2019.

68. Luo H, Pan X, Wang Q, Ye S, Qian Y. Logistic regression and random forest for effective imbalanced classification. In: *2019 IEEE 43rd Annual Computer Software and Applications Conference (COMPSAC)*. ; 2019:916-917.

69. Tanha J, Abdi Y, Samadi N, Razzaghi N, Asadpour M. Boosting methods for multi-class imbalanced data classification: an experimental review. *J Big Data*. 2020;7(70).

70. Lin E, Chen Q, Qi X. Deep reinforcement learning for imbalanced classification. *Appl Intell*. 2020;50:2488-2502.
